# Maturation of Speech-Sound ERPs in 5–6-Year-Old Children: A Longitudinal Study

**DOI:** 10.3389/fnins.2018.00814

**Published:** 2018-11-06

**Authors:** Tanja Linnavalli, Vesa Putkinen, Minna Huotilainen, Mari Tervaniemi

**Affiliations:** ^1^Cognitive Brain Research Unit, Faculty of Medicine, University of Helsinki, Helsinki, Finland; ^2^Cicero Learning, Faculty of Educational Sciences, University of Helsinki, Helsinki, Finland; ^3^Turku PET Centre, University of Turku, Turku, Finland

**Keywords:** MMN, P3a, LDN, speech, children, maturation

## Abstract

The maturation of 5–6-year-old children’s auditory discrimination – indicated by the development of the auditory event-related-potentials (ERPs) – has not been previously studied in longitudinal settings. For the first time, we present here the results based on extensive dataset collected from 75 children. We followed the 5- to 6-year-olds for 20 months and measured their ERPs four times with the same multifeature paradigm with phonemic stimuli. The amplitude of the mismatch negativity (MMN) response increased during this time for vowel, vowel duration and frequency changes. Furthermore, the P3a component started to mature toward adult-like positivity for the vowel, intensity and frequency deviants and the late discriminative negativity (LDN) component decreased with age for vowel and intensity deviants. All the changes in the components seemed to happen during the second follow-up year, when Finnish children are taught letter symbols and other preliminary academic skills before going to school at the age of seven. Therefore, further studies are needed to clarify if these changes in the auditory discrimination are purely age-related or due to increasing linguistic knowledge of the children.

## Introduction

Auditory event-related responses are an important tool to investigate auditory cognition and its development beyond behavioral measures. However, the maturation of auditory event-related responses in children is a field not well covered by the present literature. Some components – such as mismatch negativity (MMN) – are known to be evident already in new-born babies, while our knowledge of the emergence of others [e.g., P3a and late discriminative negativity (LDN)] is scarce and even contradictory. The present knowledge on components elicited by changes in the sound stream, namely MMN, P3a and LDN, is briefly presented below.

The mismatch negativity (MMN) is a component of event-related responses that is thought to reflect the neural discrimination of change in the stream of repeating stimuli (Näätänen, 1992) or a mismatch between the predicted and perceived acoustic input (Winkler et al., 2009). Apparently, in adults the MMN is of negative polarity and it is thought to originate from two main areas, namely prefrontal cortex and the supratemporal planes of the auditory cortices (Näätänen and Escera, 2000; Rinne et al., 2000).

The MMN is a very convenient tool for studying children, as it does not require concentration in task to be elicited (Näätänen et al., 2010; for a review, see e.g., Näätänen et al., 2007). Regardingdevelopmental studies, it is noteworthy that already fetuses (Huotilainen et al., 2005) and newborn babies show MMN-like responses (Cheour et al., 2000; Trainor et al., 2001; Kushnerenko et al., 2002b; Partanen et al., 2013a) for e.g., frequency changes (Alho et al., 1990), speech stimuli (Csépe, 1995), musical stimuli (Partanen et al., 2013a) and emotional pseudo-word stimuli (Kostilainen et al., 2018). Likewise, the MMN is well established in pre-schoolers (Lovio et al., 2009; Lee et al., 2012) and in schoolchildren (Kraus et al., 1999; Cheour et al., 2000; Datta et al., 2010). Yet, with subtle acoustic changes, MMN amplitudes are reported to be small during preschool and early school-age (Lovio et al., 2009; see e.g., Cheour, et al., 2000). The MMN or its early counterpart mismatch response (MMR) have been recorded in 3–12-year-old children for changes in frequency (Shafer et al., 2000; Maurer et al., 2003), intensity (Lovio et al., 2009; Lovio et al., 2010; Partanen et al., 2013c), phonemes (Kraus et al., 1999; Čeponienè et al., 2004; Lovio et al., 2009, 2010; Datta et al., 2010; Kuuluvainen et al., 2016), and vowel duration (Lovio et al., 2009, 2010). Furthermore, the MMN has been recorded in children for more abstract features, such as changes of direction of frequency change in pairs of sounds (Gumenyuk et al., 2003).

The maturation of MMN responses has been studied predominantly with cross-sectional studies. Shafer et al. (2010) did not find any difference in magnitude of the MMN amplitude between 4–5-year-old and 6–7-year-old children’s responses to vowel changes, suggesting that the amplitude does not increase during these years. In another study, Shafer et al. (2000) compared the responses for frequency changes in children and adults. There was no difference in the MMN amplitudes between the four age groups (4-year-olds, 5–6-year-olds, 7–8-year-olds, and 9–10-year-olds), or between children and adults. Gomot et al. (2000) studied the maturation of MMN components by comparing 5–7-year-old children’s, 8–10-year-old children’s and adults’ responses to frequency changes. In line with Shafer et al. (2000, 2010), there were no statistically significant differences in mean amplitudes of frontal MMN between any of the three groups.

Inconsistent with these studies, Lee et al. (2012) compared 4-, 5- and 6-year-old children, and found that small vowel deviances elicited adult-like MMN responses only in the oldest child group. For small and large lexical tone changes and large vowel changes, the MMN amplitudes were similar in all age groups. Furthermore, Bishop et al. (2011) compared 7–12-year-old children to 13–16-year-old teenagers and adults. The responses for frequency and phoneme changes revealed that the MMN amplitude increased with age. Additionally, Lovio et al. (2009) studied MMN responses of 6–7-year-old children for vowel, vowel duration, consonant, frequency and intensity change. The children’s MMN amplitudes were smaller than those observed in adults in a study by Pakarinen et al. (2009) that used the same multifeature paradigm. Partanen et al. (2013c) studied 4–6-year-old pre-schoolers’ and 7–12-year-old schoolchildren’s MMN responses to changes in vowel duration, frequency, gap, intensity and vowel identity, and found that only the older children showed MMN responses to vowel change. However, only the younger children showed MMNs to frequency deviants.

Based on the literature, it is difficult to summarize the maturation of MMN responses: Shafer et al. (2000, 2010) and Gomot et al. (2000) did not find any evidence for age-related differences in the magnitude of MMN, while other studies did (Bishop et al., 2011; Liu et al., 2014; also compare Lovio et al., 2009 vs. Pakarinen et al., 2009). The discrepant results might result from methodological differences across these studies: the paradigms are different, the saliency of changes in different deviant types (e.g., vowel change vs. consonant change) is not comparable, the age-groups might be composed of children from different developmental stages and the number of participants is mostly small, considering the amount of variance that children’s responses typically represent.

In adults studied in a passive condition, the MMN is sometimes followed by a fronto-centrally maximal positive peak with latency around 300 ms referred to as the P3a response. The P3a is thought to reflect orienting of attention (Escera et al., 1998; Friedman et al., 2001; Berti et al., 2004; Polich, 2007), and very salient or novel distractors elicit larger P3a components than more subtle ones (Escera et al., 1998; Yago et al., 2001; Berti et al., 2004). Already infants show a positive component to large deviants, similar to the adult P3a (Kushnerenko et al., 2002a; Kushnerenko et al., 2007; Háden et al., 2009). Putkinen et al. (2012) found a P3a in 2-year-old children in response to salient deviants, such as large frequency and duration changes, sound-source location deviants and novel sounds. Wetzel et al. (2006) compared the P3a responses of adults, 6–8- and 10–12-year-old children in a passive condition, and found that unlike adults, both child groups showed P3a responses to frequency deviation with younger group showing larger responses. Furthermore, Gumenyuk et al. (2004) found some age-related differences in P3a amplitudes while comparing 8–9-, 10–11- and 12–13-year-old children. The P3a response for novel sounds was significantly smaller in the oldest child group than in the younger groups that did not differ from each other. However, as Kihara et al. (2010) studied 4 to 12-year-old children, they found that P3a responses to novel sounds were larger in older children. Additionally, some studies have not found any age-related differences in P3a responses (Ruhnau et al., 2010, 2013). As the scarcity of the literature reveals, more research is needed on P3a and its maturation.

The LDN (Korpilahti et al., 1995), is a fronto-central negative response occurring typically 350-550 ms after stimulus onset, although it has been reported in later latency ranges (Putkinen et al., 2012; Ervast et al., 2015). As the LDN seems to have distinct neural generators from those of the MMN (Čeponienè et al., 2004; Hommet et al., 2009), it should not be regarded as a late manifestation of the MMN. The functional significance of LDN response is not clear: some studies have reported it to be more pronounced for speech than non-speech sounds (Korpilahti et al., 1996, 2001; Bishop et al., 2011; Kuuluvainen et al., 2016), whereas others have not found any such effect (Čeponienè et al., 2002; Putkinen et al., 2012).

The LDN response has been recorded mainly in pre-school (Korpilahti et al., 1995, 2001; Ceponiene et al., 2003; Maurer et al., 2003) and school-age children (Korpilahti et al., 1995; Čeponienè et al., 1998, Cheour et al., 2001; Čeponienè et al., 2002; Shafer et al., 2005; Hommet et al., 2009; Datta et al., 2010; Bishop et al., 2011; Liu et al., 2014). It has been reported to be nearly absent in adults (Liu et al., 2014). For instance, Gumenyuk et al. (2004), compared small groups of 8–9-, 10–11- and 12–13-year-old children and found that the youngest group had larger LDN amplitudes to novel sounds than the older groups which did not differ from each other. In line with this, Bishop et al. (2011) studied groups of 7–12-year-old children, 13–16-year-old adolescents, and adults, and found that LDN responses to phoneme deviation decreased with age. Furthermore, Hommet et al. (2009) compared the responses of 8–10-year-old children and 14–23-year-old adolescents and young adults with developmental dyslexia to those of matched peer groups without dyslexia. In typically developing participants, the younger group showed larger LDN amplitudes to consonant change. However, no such difference was found in dyslexic participants.

There is also evidence that does not support the suggestion of LDN magnitude decreasing with age. In a study comparing 3–4-year-old children, 8–9-year-old children and adults, Liu et al. (2014) found that even though both child groups showed late negativity for lexical tone change not seen in the adults, the older children showed the largest LDN responses for consonant contrasts. In addition, Hong et al. (2018) compared 6-year-old poor and typical readers and found that LDN responses for consonant changes were smaller in poor readers’ group, suggesting that larger LDN indicates more mature responses.

To summarize, we do not know how LDN matures over the childhood years, and whether its magnitude actually depends on the stimulus type.

Our aim was to monitor the maturation of auditory change-related responses, MMN, P3a and LDN in pre-school children. As low socio-economic status (SES) is known to have an association with brain activity (Tomalski et al., 2013) and reduced language and literacy skills (see e.g., Lipina and Posner, 2012), we also wanted to see whether socio-economic background of the children – represented here by maternal education level – affects the studied maturation.

The study is part of a larger project investigating children’s neural speech-sound processing and linguistic development (Linnavalli et al., 2017, 2018). The 5–6-year-olds were chosen as participants because this age-group has not been previously studied in longitudinal settings with auditory ERPs. In addition, children in Finland are given tuition in some academic skills (like recognizing the alphabet and numbers) at the age of 5–6 years in order to get them prepared to enter primary school and thus, this is a very important period for the development of linguistic skills. So, investigating their neurocognitive development is of great importance in order to know e.g., about the progress in children’s perceptual and cognitive processes in audition.

To our knowledge, this is the first study following a considerable number (75) of 5–6-year-olds for nearly 2 years. The children were measured four times with the same paradigm including phonemic changes. This experimental paradigm allows us to make strong conclusions of the maturation of children’s responses before school-age.

## Materials and Methods

### Participants

Originally 84 children were recruited from 14 municipal kindergartens to participate in the study. Five children dropped out from the EEG study after the first measurement and two were excluded because of developmental problems. Furthermore, two were excluded due to too noisy data in more than two measurements. Thus, 75 children were included in the study [mean age in the first measurements being 63 months (SD 3.2), in the second measurements 70 months (3.1), in the third 77 (3.1) and in the fourth 83 (3.2)]. The children attended municipal kindergartens in Helsinki metropolitan area, and 62 of them were native Finnish speakers. Thirteen were bilinguals having some other language than Finnish as their native language but attending Finnish-language kindergartens. The mean for mother’s education was 4.8 (1.5) on a scale from 1–7, where 5 stands for lower university/bachelor’s degree.

The guardians signed a written informed consent and the children were asked for their verbal assent before each experiment. The experiment protocol was approved by The Review Board of the Humanities and Social and Behavioral Sciences in the University of Helsinki, Finland.

### The ERP Paradigm

**FIGURE 1:**
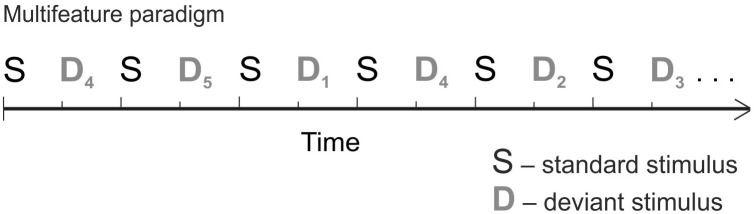
The multifeature paradigm.

The stimuli were made with semisynthetic Speech Generation Method (for details, see Alku et al., 1999). In order to collect a large amount of data in a short time – essential when measuring children – we used the multifeature paradigm (Figure 1) (Näätänen et al., 2004). In the multifeature paradigm, every other stimulus is a standard and every other a deviant, and several different deviant types alternate so that each deviant type differs from the standard in only one feature (e.g., in frequency or duration). Thus, even though the deviants occur in 50% of the sounds, each deviant type appears only in e.g., 10% of the trials. MMN responses of healthy adults and children in the multifeature paradigm have been shown to be comparable to those elicited by traditional oddball paradigm (Kujala et al., 2006; Pakarinen et al., 2009; Partanen et al., 2013b,c).

The standard stimuli STD (*P* = 0.50) were either /pi:/ or /te:/, presented in separate blocks (Table 1). As deviating stimuli, the paradigm included vowel change VOW (*P* = 0.10), vowel duration change DUR (*P* = 0.10), consonant change CON (*P* = 0.10), intensity change INT (louder *P* = 0.05 and softer *P* = 0.05) and frequency change FRE (higher *P* = 0.05, lower *P* = 0.05). The duration of all stimuli was 170 ms, excluding vowel duration change DUR (100 ms). Stimulus onsets were 500 ms apart from each other. F0 was 101 Hz, excluding the frequency change FRE which had the f0s of either 93 or 109 Hz. Intensity of the stimuli was ∼70 dB (SPL), and the intensity change INT had intensities of either 63 or 77 dB. There were 465 stimuli in each of the four blocks that were counterbalanced. Each block lasted for about 5 min and the total EEG recording net time was 20 min. Only the participants with four accepted blocks from the measurement were included in the analyses. The identical experiment paradigm has been used in measuring MMN responses in adults (Pakarinen et al., 2009) and children (Lovio et al., 2009; Linnavalli et al., 2017).

**Table 1:** The stimuli of the multifeature paradigm.

Block	STD	VOW	DUR	CON	INT	FRE
1 & 2	/te:/	/ti:/	/te/	/pe:/	± 7 dB	± 8%
3 & 4	/pi:/	/pe:/	/pi/	/ti:/	± 7 dB	± 8%

*Four blocks (two blocks for each standard stimuli) were played for the participants. The blocks were played in randomized order*.

### The Procedure

All the EEG measurements were conducted during the children’s normal daily stay at kindergarten in the kindergarten premises, in separate rooms with only the participant and the experimenter(s) present. During the measurement, the children watched a muted children’s movie, and were asked to avoid unnecessary movement, to ignore the experimental stimuli, and to concentrate on the movie. The stimuli were presented via Sony Professional MDR-7506 headphones. Cookies and soft drinks were offered during the short breaks between blocks. With preparation, one measurement took approximately an hour.

### Data Recording and Processing

The experimental paradigm was implemented with *Presentation 17.0* (Neurobehavioral Systems, Inc., Albany, CA, United States). The EEG was recorded with 32 Ag-AgCl scalp electrodes according to international 10–20 system by using ActiCap (Brain Products, Germany). The EEG equipment was portable (Brainvision QuickAmp amplifier). The EEG data were registered with sample rate of 500 Hz. Recording reference was the average signal of all electrodes. Two additional active electrodes were placed on the mastoid bones.

EEG was processed with BESA 5.3. software (MEGIS Software GmbH, Gräfelfing, Germany). We interpolated noisy electrodes and removed eye blink artifacts using semi-automatic Besa PCA method. The percentage of accepted trials averaged over all participants and the number of interpolated channels averaged over all blocks for each measurement are listed in Table 2. Frequencies under 0.5 Hz and over 30 Hz were filtered out offline and we re-referenced the data to the mean of the mastoids. Inspected epochs were extracted from EEG from -100 ms before onset to 500 ms after the onset of the stimuli. EEG-epochs with amplitudes exceeding ± 120 μV were excluded from the analyses. The responses were averaged for each participant and the averaged responses were then exported to MATLAB R2017 (The MathWorks Inc., Natick, MA, United States).

**Table 2:** The percentage of accepted trials and the number of interpolated channels.

Measurements				
	1st (*N* = 74)	2nd (*N* = 66)	3rd (*N* = 61)	4th (*N* = 65)
*Accepted trials (percentage)*			
mean (SD)	93.3 (5.9)	93.6 (5.3)	92.7 (6.1)	95.0 (4.6)
max	99.3	99.2	99.2	99.3
min	67.0	67.1	69.0	66.5
median	94.8	95.5	93.8	96.1
*Interpolated channels (number)*				
**Out of 32 channels**				
mean (SD)	2.0 (1.5)	2.4 (1.8)	2.2 (1.4)	2.1 (1.4)
max	7	8	8	7
min	0	0	0	0
median	1	2	2	2
**Out of 9 channels**				
mean (SD)	0.69 (0.71)	0.72 (0.71)	0.59 (0.65)	0.43 (0.58)
max	3	3	3	3
min	0	0	0	0
median	1	1	1	0

*The accepted trials are averaged over all participants and interpolated channels over all blocks (4 blocks per a participant) for each measurement. The interpolated channels are reported for both all 32 channels used in the measurements and for 9 channels (F3, Fz, F4, C3, Cz, C4, P3, Pz, and P4) used in the analyses*.

For the intensity deviant, we averaged together the responses to both intensity changes (louder and softer) and similarly, for the frequency deviant response we averaged together the responses to increments and decrements of frequency. The standard and deviant trials from all four blocks were combined according to their stimulus category. The subtraction signals were created for each deviant stimulus by subtracting the participant’s average standard response from the average deviant responses, separately for each participant, each deviant, and each electrode. Typically for MMN paradigms, we inspected further the electrodes F3, Fz, F4, C3, Cz, C4, P3, Pz and P4, as this reveals front-back and left-right distribution of the brain responses. Mean amplitudes were calculated separately for each deviant and each measurement for MMN, LDN and P3a responses over 50 ms time window. The time windows were chosen based on visual inspection supported by data from previous studies showing children’s MMN, LDN and P3a responses.

### Statistical Analyses

Several children either did not participate in all four measurements or showed noisy data that had to be rejected, on some measurement points. Thus, the number of participants varied in the measurements, being 74 in the first, 66 in the second, 61 in the third and 65 in the fourth measurement. Therefore, we conducted the analyses with linear mixed models, more specifically, with *linear growth curve model* (West, 2009) that allows the analysis of longitudinal data with different number of data points per subject. In addition, with linear growth curve model it is possible to take into account the individual ages of each participant during each measurement. The analyses were run separately for all inspected responses. Centered values for age (months) and mother’s education (scale from 1–7), along with all the interaction between these, acted as predictors and averaged responses as dependent variables. For MMN and P3a, we averaged the responses for each deviant over the frontline electrodes (F4, Fz, and F3) and for LDN we averaged the responses for each deviant over all the nine electrodes (F4, Fz, F3, C3, Cz, C4, P3, Pz, and P4).

We used random intercept model in all analyses and chose compound symmetry as the covariance structure on the basis of Schwarz’s Bayesian Criterion (BIC). We conducted the analyses with SPSS 24 (IBM Corporation, NY, United States) and set the alpha level at *p* < 0.05.

## Results

All the averaged MMN and LDN responses over chosen electrodes were significantly different from zero at the group level in the inspected time windows (*p* < 0.001, each). The P3a response was significantly different from zero at the group level in the inspected time windows (*p* < 0.001, each), excluding the vowel duration deviant in all of the four measurements (*p* = 0.665, *p* = 0.256, *p* = 0.764 and *p* = 0.342, respectively) and the frequency deviant in the fourth measurement (*p* = 0.055). Mean amplitudes and peak latencies defining the inspected time windows for each response, deviant and measurement are depicted in Tables 3, 4.

**Table 3:** Mean MMN, P3a and LDN (μV) amplitudes for all four measurements.

Amplitude (SD) μV					
Measurement		1st	2nd	3rd	4th
*MMN*					
	VOW	-1.97 (2.6)	-2.58 (2.6)	-3.38 (3.0)	-2.89 (2.8)
	DUR	-3.65 (2.4)	-4.14 (2.9)	-4.83 (3.1)	-4.58 (2.4)
	CON	-2.32 (2.2)	-2.75 (2.5)	-2.78 (2.5)	-2.63 (2.1)
	INT	-2.62 (2.4)	-2.89 (2.2)	-2.26 (2.5)	-2.42 (2.0)
	FRE	-1.98 (2.5)	-2.56 (2.6)	-3.05 (2.9)	-2.63 (2.8)
*P3a*					
	VOW	-4.37 (2.6)	-3.99 (2.8)	-3.44 (2.9)	-1.42 (2.9)
	DUR	-0.15 (3.0)	-0.32 (2.3)	-0.09 (2.5)	-0.22 (1.9)
	CON	-3.08 (2.3)	-3.27 (2.6)	-3.76 (2.4)	-3.19 (2.3)
	INT	-3.02 (2.5)	-3.56 (2.4)	-3.34 (2.3)	-2.27 (2.0)
	FRE	-2.51 (2.2)	-2.52 (2.4)	-2.04 (2.6)	-0.65 (2.7)
*LDN*					
	VOW	-5.63 (2.7)	-5.83 (3.2)	-6.08 (2.8)	-5.25 (2.5)
	DUR	-1.67 (2.4)	-1.76 (2.2)	-2.01 (2.1)	-1.70 (1.9)
	CON	-3.45 (2.2)	-3.64 (2.4)	-4.00 (2.4)	-3.04 (2.2)
	INT	-3.71 (2.1)	-3.93 (2.4)	-3.73 (2.2)	-2.94 (2.2)
	FRE	-3.86 (2.3)	-3.55 (2.3)	-4.35 (2.1)	-3.38 (2.3)

*Mean amplitudes for the MMN and the P3a are averaged over F3, Fz, and F4 electrodes and for the LDN over F3, Fz, F4, C3, Cz, C4, P3, Pz, and P4 electrodes*.

**Table 4:** The MMN, P3a and LDN peak latencies in milliseconds from the stimulus onset for each deviant in each measurement.

Latency (ms)					
Measurement		1st	2nd	3rd	4th
*MMN*					
	VOW	173	173	177	164
	DUR	249	245	241	238
	CON	299	297	267	290
	INT	306	297	257	242
	FRE	273	267	249	242
*P3a*					
	VOW	339	345	327	329
	DUR	323	319	319	319
	CON	361	351	361	363
	INT	333	339	335	329
	FRE	361	357	339	357
*LDN*					
	VOW	473	457	473	473
	DUR	397	393	397	385
	CON	473	461	447	467
	INT	473	469	473	473
	FRE	473	439	473	473

*Please note that for the duration deviant, the change occurs at 100 ms after stimulus onset*.

**FIGURE 2:**
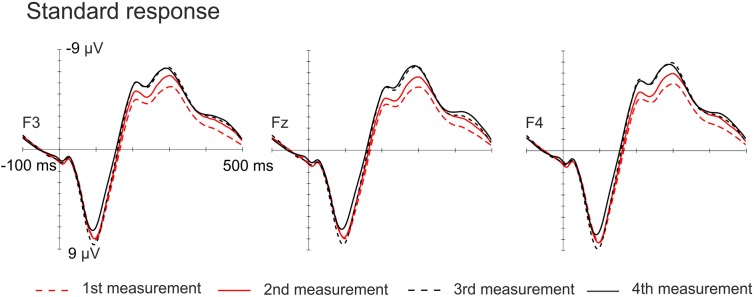
Standard responses on frontline electrodes for all four measurements.

**FIGURE 3:**
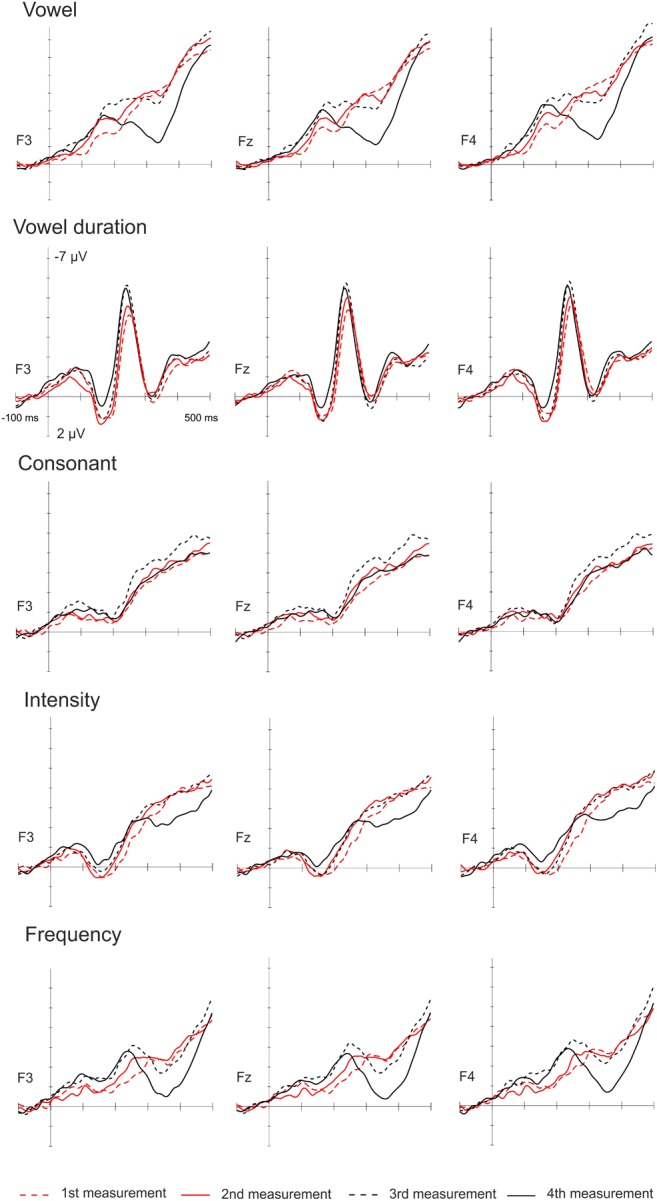
Subtraction signals on frontline for all four measurements for all deviants.

The amplitudes of the MMN responses for frequency, vowel duration and vowel deviants increased with age, whereas the amplitudes decreased in the P3a time-window for frequency, vowel and intensity deviants. The amplitudes of the LDN responses for vowel and intensity deviants decreased by age during the follow-up. All the averaged standard responses are depicted in Figure 2 and all the subtraction signals for frontline electrodes are depicted in Figure 3. We report the significant or near significant main effects and interactions in the results. Additional tables covering all the results are in Supplementary Information.

Parameter estimates indicate how many *microvolts* the inspected responses change when variables with significant or marginally significant main effects or interactions increase one step (months for age and steps on a scale from 1 to 7 for mother’s education).

### MMN

The main effect of age was significant on the front line electrodes for *vowel deviant* [*F*(1,191) = 9.810, *p =* 0.002, parameter estimate -0.052988], *duration deviant* [*F*(1,198) = 11.337, *p =* 0.001, parameter estimate -0.059168] and *frequency deviant* [*F*(1,189) = 5.285, *p =* 0.023, parameter estimate -0.034871], showing an increase in MMN responses with age. All the results are depicted in Supplementary Table S1.

### P3a

**FIGURE 4:**
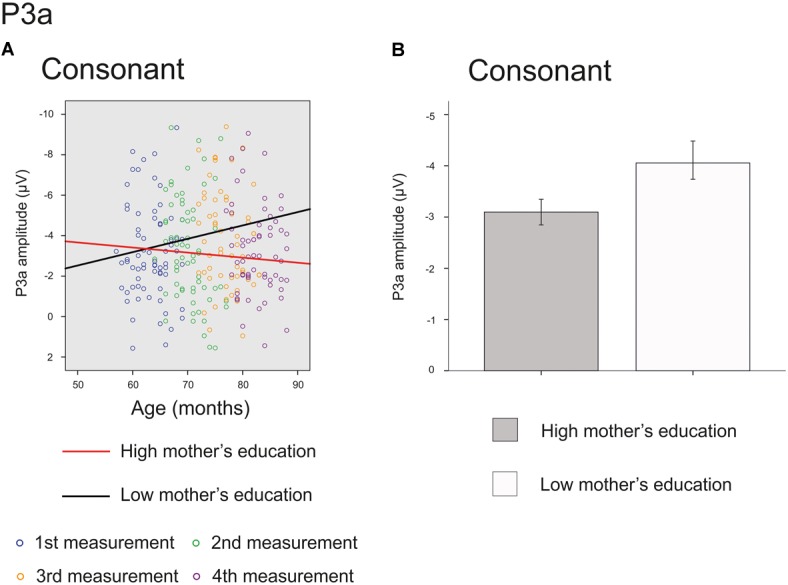
Significant interaction and marginally significant main effect for P3a response. The cut-off points for mother’s education in figures are for illustration purposes only. **(A)** Individual P3a amplitudes for *consonant deviants* for all four measurements. Red line represents change in amplitudes for an individual with high (6/7) and black line for low (2/7) maternal education. **(B)** P3a amplitudes for *consonant deviant* averaged over all measurements for children with high (6/7) or low (2/7) maternal education (High: mean amplitude –3.10 μV, SEM = ± 0.28, Low: mean amplitude –4.06 μV, SEM ± 0.40).

The main effect of age was significant on the front line electrodes for *vowel deviant* [*F*(1,214) = 46.864, *p <* 0.001, parameter estimate 0.140570], *intensity deviant* [*F*(1,210) = 4.692, *p =* 0.031, parameter estimate 0.037629] and *frequency deviant* [*F*(1,209) = 24.889, *p <* 0.001, parameter estimate 0.089703], showing a decrease in amplitude in inspected time window. Furthermore, there was a significant interaction of mother’s education and age for *consonant deviant* [*F*(1,206) = 3.937, *p =* 0.049, parameter estimate 0.022674] (Figure 4A) and a trend for the main effect of mother’s education for *consonant* deviant [*F*(1,66) = 3.167, *p =* 0.080 parameter estimate 0.236972] (Figure 4B), indicating that the P3a responses of children with higher maternal education were in average more mature and matured more with age than those of their peers with lower maternal education. All the results are depicted in Supplementary Table S2.

### LDN

**FIGURE 5:**
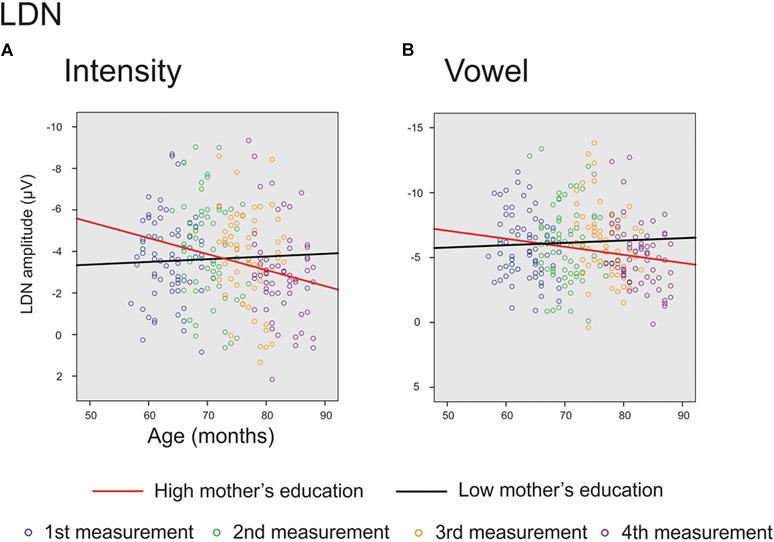
Significant and marginally significant interactions for LDN response. The cut-off points for mother’s education in figures are for illustration purposes only. The red line represents change in amplitudes for an individual with high (6/7) and the black line for an individual with low (2/7) maternal education. **(A)** Individual LDN amplitudes for *intensity deviant* for all four measurements. **(B)** Individual LDN amplitudes for *vowel deviant* for all four measurements.

The main effect of age was significant on averaged deviants over nine electrodes for *intensity deviant* [*F*(1,201) = 8.220, *p =* 0.005, parameter estimate 0.042873] and marginally significant for *vowel deviant* [*F*(1,195) = 3.701, *p =* 0.056, parameter estimate 0.033238], showing a decrease in LDN responses with age. In addition, there was a significant interaction of age and mother’s education for *intensity* [*F*(1,201) = 4.839, *p =* 0.029, parameter estimate 0.021813], indicating that higher mother’s education further decreased the mean LDN amplitudes for *intensity* change (Figure 5A). Additionally, there was a trend for the interaction of age and mother’s education for *vowel* deviant [*F*(1,195) = 2.922, *p =* 0.089, parameter estimate 0.019586], indicating that these responses decreased more with age in children with higher maternal education (Figure 5B). All the results are depicted in Supplementary Table S3.

## Discussion

To our knowledge, this is the first auditory ERP study reporting such a large number of children followed for nearly 2 years and measured four times with the paradigm including five deviants in parallel. In addition, instead of treating each measurement time as representative of mean age of children, our statistical analysis allowed us to take each individual measurement age into account, contributing to a model describing more accurately the development during the inspected 20 months.

### MMN

Responses elicited by the *vowel deviant*, the *vowel duration deviant* and the *frequency deviant* increased clearly over the studied 20 months suggesting that at least during the 5th and 6th years of life, auditory change detection is still enhancing. Instead, there seems to be no signs of increase in MMN components for *consonant* and *intensity deviants.* The change in *consonant deviant* occurs within the first tens of milliseconds, and appears not to be very salient. Consistently with our results, a study in adults did not show any prominent MMN – not to mention P3a – peaks in a similar paradigm used by Pakarinen et al. (2009). As for the *intensity deviant*, the mean MMN amplitudes are rather large compared with other deviants already in the beginning of the follow-up and this might explain why no increase was detected.

There is contradictory evidence regarding the increase or decrease of MMN amplitude with age and it is not known if MMN maturation has different phases along the childhood. According to our study, with 75 same-age participants, it seems that during the ages 5–6 years the pre-attentive auditory change detection for phoneme changes is still developing. What happens after this age-range, is not known and more longitudinal research is needed.

### P3a

The stimulus changes in our study were acoustically small, and this probably explains why positive P3a was not apparent in any of the responses. However, the P3a responses elicited by the *vowel deviant, intensity deviant* and the *frequency deviant* changed over the studied 20 months. Thus, it seems that orienting of attention, which P3a is thought to reflect, is enhancing during the inspected 2 years for these sound features.

The P3a elicited by the *vowel duration deviant* did not change with age, and judging by the shape, the response to this deviant seems to be very solid early on. Apparently, duration change being very salient even for pre-schoolers might be due to Finnish being a quantity language and children learning to differentiate words based on the duration of phonemes (both vowel and consonant lengths) already at the early stage of life. Larger MMN responses of Finnish speakers to phoneme duration have been shown in adults (Ylinen et al., 2005; Tervaniemi et al., 2006) and are likely to occur already in childhood. Another possible explanation is that – unlike changes in phonemes – processing duration does not require analysis of any subtle changes in sound features but simply noticing whether there is an ongoing sound or not. Still, whereas adults in similar paradigm (Pakarinen et al., 2009) showed a positive peak around 300 ms from sound onset, the detected deviation in duration does not seem to be large enough to elicit a positive P3a response already in 5–6-year-old children. Thus, the orienting of attention – while clearly more salient for duration change than for other deviants – will continue to develop later in childhood or adolescence.

While the main effect of age was not significant for the P3a responses for *consonant deviant*, there was a significant interaction of age and mother’s education and a marginally significant main effect of mother’s education for this response. These results suggest that the higher-SES children’s P3a responses mature slightly faster than those of their lower-SES peers for consonant deviant during the inspected 20 months. Interestingly, based on the estimated slopes visualized in Figure 4A, it seems that the responses of children with lower-SES do not approach the positive values but show the opposite trajectory and grow more negative with age. This result is difficult to interpret but could be due to many ongoing processes of ERP maturation or differences in component latencies between low-SES and high-SES children. Some previous studies (Gumenyuk et al., 2004; Wetzel et al., 2006) have found larger P3a responses in younger compared to older children, but our results do not support these findings. Naturally, it could be that the neural orienting of attention is going through different phases – P3a response increasing and decreasing – before auditory discrimination system is complete.

### LDN

The LDN responses decreased significantly with age for the *intensity deviant* and marginally for the *vowel deviant*. Furthermore, the LDN amplitude for *intensity deviant* and marginally also for *vowel deviant* decreased with age more in children with higher maternal education. These results are in line with previous studies (Gumenyuk et al., 2004; Hommet et al., 2009; Bishop et al., 2011) showing smaller LDN amplitudes for older children, and further suggest that higher SES is connected with faster maturation of LDN responses. However, we did not find any support for the suggestion that LDN is related specifically to language processing (Korpilahti et al., 1996, 2001; Bishop et al., 2011; Kuuluvainen et al., 2016). Of course, this interpretation is questionable since all the stimuli were basically linguistic. Nevertheless, as the frequency and the intensity changes do not convey any linguistic meaning in Finnish language (except emotional connotations of the speaker), one would assume that the maturation of LDN responses would prove different for vowel, vowel duration and consonant changes compared to these, if the response was merely related to linguistic functions.

## Conclusion

The variation in children’s individual responses is large, and a lot of information is lost in averaging responses over fixed time window. Nevertheless, based on our study it seems clear that the auditory event-related potentials reflect changes in the processing of speech sounds between 5 and 6 years of age, showing enhancement and gaining of accuracy for several speech sound features. In connection with some speech-sound features, higher SES appears to boost this maturation. However, SES does not seem to have a profound effect on the maturation of these responses, which could be explained in our study by our sample of kindergarten children. Irrespective of the location, Finnish municipal kindergartens are of similar high quality with teachers having an academic degree. Furthermore, the kindergartens are low-cost (free for low-SES families), which allows children from different socio-economic backgrounds to benefit from early childhood education.

In future studies, the contribution of the teaching of letter symbols in pre-school (starting properly at the age of six in Finland) on the development of the neural speech-sound discrimination needs to be investigated. Furthermore, taking into account our recent findings about the correspondence between individual linguistic skills and MMN attributes in pre-school children (Linnavalli et al., 2017), in future one might be able to investigate a child’s linguistic development with event-related potentials and thus identify the individuals benefiting from interventions enhancing language skills or auditory attention.

## Data Availability

The pre-processed and epoched EEG data supporting the conclusions of this manuscript will be made available on reasonable request addressed to the authors, without undue reservation, to any qualified researcher.

## Ethics Statement

This study was carried out in accordance with the Declaration of Helsinki and The Review Board of the Humanities and Social and Behavioral sciences in the University of Helsinki. We obtained written informed consent from the guardians of all subjects and verbal assent from the children participating in the experiments. The protocol was approved by The Review Board of the Humanities and Social and Behavioral sciences in the University of Helsinki, Finland.

## Author Contributions

TL, MT, and MH designed the experiments. TL collected the data. TL and VP conducted the analyses. TL, VP, MH, and MT wrote the paper.

## Conflict of Interest Statement

The authors declare that the research was conducted in the absence of any commercial or financial relationships that could be construed as a potential conflict of interest.
