# Supplementary material for: Maturation of Speech-Sound ERPs in 5–6-Year-Old Children: A Longitudinal Study
Source: Front Neurosci. 2018 Nov 6;12:814. doi: 10.3389/fnins.2018.00814 (PMC6232289; doi:10.3389/fnins.2018.00814)
Supplement: Supplementary file 1 [file Table_1.DOCX]

**Supplementary information**

**Table S1** The main effects and interactions for all deviants’ mismatch negativity (MMN) mean amplitudes in front line [(F3+Fz+F4)/3]. The significant results are marked in bold.

|  |  | *F* | *p* |
| --- | --- | --- | --- |
| *Vowel* | |  |  |
|  | *Age* | *F(1, 191)*=9.810 | ***p=*.002** |
|  | *Mother’s education* | *F(1,64)=*.282 | *p=*.597 |
|  | *Mother’s education x Age* | *F(1,190)=*.294 | *p=*.588 |
| *Vowel duration* | |  |  |
|  | *Age* | *F(1, 198)=*11.337 | ***p=*.001** |
|  | *Mother’s education* | *F(1,63)=*1.406 | *p=*.240 |
|  | *Mother’s education x Age* | *F(1,198)=*1.519 | *p=*.219 |
| *Consonant* | |  |  |
|  | *Age* | *F(1, 206)=*.305 | *p=*.582 |
|  | *Mother’s education* | *F(1,64)=*.687 | *p=*.410 |
|  | *Mother’s education x Age* | *F(1,206)=*1.557 | *p=*.214 |
| *Intensity* | |  |  |
|  | *Age* | *F(1, 207)=*1.767 | *p=*.185 |
|  | *Mother’s education* | *F(1,64)=*.907 | *p=*.344 |
|  | *Mother’s education x Age* | *F(1,208)=*.011 | *p=*.918 |
| *Frequency* | |  |  |
|  | *Age* | *F(1, 189)*=5.285 | ***p=*.023** |
|  | *Mother’s education* | *F(1,63)=*.817 | *p=*.369 |
|  | *Mother’s education x Age* | *F(1,189)=*.171 | *p=*.680 |

**Table S2** The main effects and interactions for all deviants’ P3a mean amplitudes in front line [(F3+Fz+F4)/3]. The significant and marginally significant results are marked in bold.

|  |  | *F* | *p* |
| --- | --- | --- | --- |
| *Vowel* | |  |  |
|  | *Age* | *F(1, 214)*=46.864 | ***p<*.001** |
|  | *Mother’s education* | *F(1,59)=*.076 | *p=*.784 |
|  | *Mother’s education x Age* | *F(1,216)=*1.193 | *p=*.276 |
| *Vowel duration* | |  |  |
|  | *Age* | *F(1,203)=*.043 | *p=*.835 |
|  | *Mother’s education* | *F(1, 66)=3.167* | *p=*.609 |
|  | *Mother’s education x Age* | *F(1,204)=*.075 | *p=*.785 |
| *Consonant* | |  |  |
|  | *Age* | *F(1,206)=*.171 | *p=*.679 |
|  | *Mother’s education* | *F(1, 66)*=3.167 | ***p=*.080** |
|  | *Mother’s education x Age* | *F(1, 206)*= 3.937 | ***p=*.049** |
| *Intensity* | |  |  |
|  | *Age* | *F(1, 210)*= 4.692 | ***p=*.031** |
|  | *Mother’s education* | *F(1,65)=* 1.648 | *p=*.204 |
|  | *Mother’s education x Age* | *F(1,212)=*.076 | *p=*.783 |
| *Frequency* | |  |  |
|  | *Age* | *F(1, 209)*=24.889 | ***p<*.001** |
|  | *Mother’s education* | *F(1,62)=*.553 | *p=*.355 |
|  | *Mother’s education x Age* | *F(1,211)=*1.443 | *p=*.231 |

**Table S3** The main effects and interactions for all deviants’ late discriminative negativity (LDN) mean amplitudes for average over nine electrodes [(F3+Fz+F4+C3+Cz+C4+P3+Pz+P4)/9]. The significant and marginally significant results are marked in bold.

|  |  | *F* | *p* |
| --- | --- | --- | --- |
| *Vowel* | |  |  |
|  | *Age* | *F(1, 195)*=3.701 | ***p=*.056** |
|  | *Mother’s education* | *F(1,66)=*.677 | *p=*.414 |
|  | *Mother’s education x Age* | *F(1, 195)*=2.922 | ***p=*.089** |
| *Vowel duration* | |  |  |
|  | *Age* | *F(1, 206)=*.027 | *p=*.869 |
|  | *Mother’s education* | *F(1,62)=*.000 | *p=*1.000 |
|  | *Mother’s education x Age* | *F(1,207)=*.683 | *p=*.410 |
| *Consonant* | |  |  |
|  | *Age* | *F(1, 207)=*1.146 | *p=*.286 |
|  | *Mother’s education* | *F(1,64)=* 1.504 | *p=*.225 |
|  | *Mother’s education x Age* | *F(1,207)=*2.272 | *p=*.133 |
| *Intensity* | |  |  |
|  | *Age* | *F(1, 201)*=8.220 | ***p=*.005** |
|  | *Mother’s education* | *F(1,65)=*.001 | *p=*.971 |
|  | *Mother’s education x Age* | *F(1, 201)*=4.839 | ***p=*.029** |
| *Frequency* | |  |  |
|  | *Age* | *F(1, 203)=*2.405 | *p=*.123 |
|  | *Mother’s education* | *F(1,64)=*.365 | *p=*.548 |
|  | *Mother’s education x Age* | *F(1,203)=*1.871 | *p=*.173 |
